# Microwave Detection of a Metal Peroxide and Two New Metal Dicarbides: The Precise Semiexperimental Equilibrium Structures of CaO_2_, SrC_2_, and YbC_2_

**DOI:** 10.1021/acs.jpca.5c07233

**Published:** 2025-12-17

**Authors:** P. Bryan Changala, Harshal Gupta, Shawn Meyer, Michael C. McCarthy

**Affiliations:** † JILA, 172322 University of Colorado Boulder and National Institute for Standards and Technology, Boulder, Colorado 80309, United States; ‡ Department of Physics, 734222 University of Colorado Boulder, Boulder, Colorado 80309, United States; § 1877 Center for Astrophysics | Harvard & Smithsonian, Cambridge, Massachusetts 02138, United States; ∥ 61814 National Science Foundation, Alexandria, Virginia 22314, United States; ⊥ Department of Electrical, Computing & Energy Engineering, University of Colorado Boulder, Boulder, Colorado 80309, United States

## Abstract

We report the pure rotational spectra of three alkaline earth­(-like) metal-bearing molecules: calcium peroxide (CaO_2_), strontium dicarbide (SrC_2_), and ytterbium dicarbide (YbC_2_), produced in a laser ablation–electric discharge supersonic expansion source and detected by cavity Fourier transform microwave spectroscopy. The semiexperimental equilibrium structure of each molecule has been derived to ≲1 mÅ uncertainty by combining comprehensive isotopic measurements with highly accurate *ab initio* rovibrational corrections. These precise structures provide direct physical probes of not only the highly ionic metal–ligand bonding but also the electronic structure of the dianionic ligands themselves. Our detection of CaO_2_, in particular, appears to represent the only high-resolution gas-phase spectroscopy of a metal peroxide (M^2+^O_2_^2–^) molecule, providing a unique intramolecular proxy of the unstable O_2_^2–^ dianion. In addition to these fundamental insights, we discuss our observation of highly nonequilibrated vibrational population distributions in the expansion source and the relevance of our results to the chemistry of circumstellar envelopes studied by radio astronomy.

## Graphical abstract

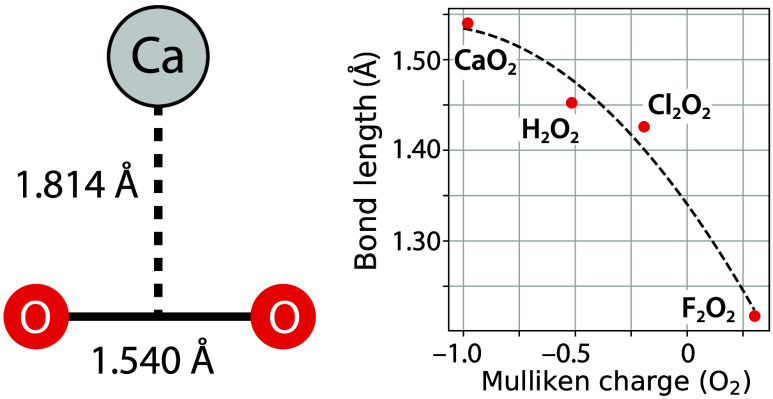

## Introduction

The alkaline earth monoxides (MO, M = Be, Mg, Ca, Sr, Ba) are among the most familiar ionic solids. They provide a template for understanding other alkaline earth compounds containing a small, closed-shell L^2–^ anion that replaces O^2–^. Such ML “pseudo-oxides”, which include some metal dicarbides and peroxides (L = C_2_, O_2_),^1,2^ have solid-state chemical and structural properties similar to those of their MO analogues. Quantum chemical theoretical studies of MO, MC_2_, and MO_2_ systems^3,4^ have demonstrated that the similarities in their bulk properties extend down to isolated molecular properties, i.e., between the structural and electronic properties of individual molecular ML monomers. Although experimental evidence for these molecular-scale parallels exists for a number of transition metal dicarbides,^5–7^ there is a general lack of spectroscopic data for the alkaline earth MC_2_ species in the gas phase, with the notable exception of the anion photodetachment spectrum of BeC_2_,^8^ and, apparently, for any metal peroxide.

These fundamental physical and chemical considerations, as well as the relevance of the closely related alkaline earth monoacetylides (MCCH) to molecular physics applications^9^ and astrochemistry,^10^ have motivated our recent interest in the spectroscopy and molecular properties of the dicarbides and peroxides of alkaline earth and alkaline earth-like metals. (We refer to a metal such as Yb as alkaline earth-like because its valence 4f^14^6s^2^ electron configuration has a closed f shell, and it participates in ionic bonding similarly as true alkaline earths by donation of s electrons.) We recently detected the pure rotational microwave spectra of gas-phase MgC_2_ and CaC_2_ in the laboratory with laser ablation–electric discharge supersonic expansion techniques. We used these data to detect both molecules in space with radio astronomy^11,12^ and, in combination with high-accuracy quantum chemical calculations, to derive their precise semiexperimental equilibrium structures. These results showed that MgC_2_ and CaC_2_ are T-shaped—by analogy with other p- and d-block metal dicarbides whose pure rotational spectra are known, including AlC_2_, GeC_2_, ScC_2_, YC_2_, and SiC_2_ ^6,7,13–15^ —and corroborated their description as pseudo-oxides with a nominal M^2+^C_2_^2–^ charge configuration.

In this paper, we further explore these electronic and structural trends in pseudo-oxides with the laboratory microwave detection of SrC_2_, YbC_2_, and CaO_2_. These systems allow us to extend our data set to heavier alkaline earth metals, alkaline earth metal-like rare earth elements, and other closed-shell L^2–^ ligands, i.e., the peroxide anion, O_2_^2–^. We report the cm-wave transition rest frequencies of the parent molecules along with several isotopic and vibrational satellites, the latter of which allow us to quantify the highly nonequilibrated vibrational temperatures in our supersonic expansion source. New follow-up measurements of vibrationally excited CaC_2_ are also reported here. We present rovibrational calculations based on coupled cluster electronic potential energy surfaces to verify our assignments and derive precise semiexperimental equilibrium structures. Our results confirm the remarkably similar structural and ionic bonding patterns of SrC_2_ and YbC_2_ with respect to MgC_2_, CaC_2_, and other metal dicarbides. Our detection of CaO_2_ is of particular interest because it appears to be the only metal peroxide for which precise gas-phase structural data are now available, allowing us to compare the O–O bond length of its nominal O_2_^2–^ ligand to peroxides and other charge states of molecular O_2_.

## Experimental Section

SrC_2_, YbC_2_, and CaO_2_ were produced in a laser ablation–electric discharge jet expansion source previously used to study a number of other metal-bearing oxides and carbides including TiO_2_ and Ge-carbon clusters and chains.^14,16,17^ The optimal source conditions were nearly identical to those from our recent studies of the closely related alkaline earth metal–carbon chains (CaCCH, SrCCH, MgCCH, MgC_4_H, and MgC_3_N^9,18^ ) and metal dicarbides (MgC_2_ ^11^ and CaC_2_ ^12^). A dilute mixture of a carbon (0.1% C_2_H_2_) or oxygen (0.2% O_2_) source in neon exited a solenoid valve backed by 2500 Torr in ∼600 μs pulses at a repetition rate of 5 Hz. A continuously rotating Sr, Yb, or Ca metallic-rod target was placed ca. 1 cm outside the valve, where it was ablated with a 20–50 mJ pulse of 532 nm radiation produced from second-harmonic generation of a 10 ns Q-switched Nd:YAG laser. The ablated metal atoms were entrained by the gas pulse, which then passed through two copper ring electrodes biased to a potential difference of 700–800 V, which produced a dc discharge with a peak current of 20–40 mA. After exiting the discharge region, the gas mixture supersonically expanded into a large vacuum chamber and cooled to a rotational temperature of a few K. The vibrational cooling in the expansion can be far less efficient for small molecules like triatomics, and as shown below, we detected vibrationally excited states with internal energies exceeding *E*/*k*_B_ = 3000 K.

The pure rotational spectrum of each target molecule was measured using a cavity Fourier transform microwave (FTMW) spectrometer operating from 5 to 26 GHz.^19,20^ The cavity axis was aligned parallel to the supersonic jet expansion resulting in two Doppler components symmetrically offset about the rest frequency of each microwave transition, which was measured with an uncertainty of 2 kHz. Additional transitions above 26 GHz were detected via double resonance (DR) depletion measurements by exciting the molecules with a microwave horn oriented perpendicular to the cavity axis. The microwave signal from SrC_2_ and YbC_2_ was sufficiently intense to observe the ^86^Sr, ^87^Sr, ^88^Sr, ^170^Yb, ^171^Yb, ^172^Yb, ^173^Yb, ^174^Yb, and ^176^Yb isotopes in natural abundance. However, only the most abundant calcium isotope (^40^Ca, 97%) was observed owing to the weaker signal from CaO_2_. Samples of statistical H^12^C^13^CH synthesized in our laboratory were used to produce ^13^C-substituted species, while commercial samples of ^18^O_2_, ^17^O_2_, and statistical ^16^O^18^O were used to produce ^17,18^O-substituted CaO_2_.

## Theory

The laboratory searches for SrC_2_, YbC_2_, and CaO_2_ were guided by theoretical predictions derived from coupled cluster theory including single, double, and perturbative triple excitations (CCSD­(T))^21,22^ as implemented in the CFOUR program package.^23,24^ The equilibrium geometry of CaO_2_ was optimized with standard analytic gradient techniques^25,26^ using the correlation-consistent polarized core–valence double-ζ to quintuple-ζ basis sets (cc-pCV*X*Z, X = D, T, Q, 5)^27,28^ correlating all electrons except Ca 1s–2p. The heavy elements Sr and Yb required effective core potentials (ECP) or relativistic treatments based on spin-free exact two-component theory in its one-electron variant (SFX2C-1e).^29,30^ For SrC_2_, we applied ECP calculations using the 28-electron relativistic core potential (ECP28MDF^31^) and corresponding pseudopotential correlation-consistent core–valence basis sets (cc-pCV*X*Z-PP),^32^ as well as SFX2C-1e-CCSD­(T) theory with weighted core–valence basis sets for Sr up to quadruple-ζ (cc-pwCV*X*Z-X2C, X = D, T, Q)^32^ and unweighted core–valence basis sets for C (cc-pCV*X*Z-X2C). For YbC_2_, only SFX2C-1e-CCSD­(T) calculations were performed with the analogous cc-pwCV*X*Z-X2C basis sets for Yb.^33^ Additional high-order correlation corrections for CaO_2_ were estimated using CCSDT­(Q)_Λ_ theory.^34–36^ The optimized theoretical geometries are summarized in Tables 1, 2, and 3.

**Table 1:** Theoretical Equilibrium Geometry and Electric Dipole Moment of SrC_2_

ECP-CCSD(T)	*r*(C–C) (Å)	*r*(Sr–C_2_) (Å)	μ (D)
cc-pCVDZ(-PP)	1.2932	2.3135	11.86
cc-pCVTZ(-PP)	1.2732	2.2675	11.86
cc-pCVQZ(-PP)	1.2695	2.2532	11.83
cc-pCV5Z(-PP)	1.2687	2.2485	11.82
CBS[T-5]^a^	1.2684	2.2463	
SFX2C-1e-CCSD(T)			
cc-p(w)CVDZ	1.2949	2.3036	11.65
cc-p(w)CVTZ	1.2771	2.2584	11.70
cc-p(w)CVQZ	1.2750	2.2495	11.73
CBS[D-Q]^b^	1.2747	2.2473	

a Complete basis-set estimate by exponential extrapolation of the TZ, QZ, and 5Z geometries.

b Complete basis-set estimate by exponential extrapolation of the DZ, TZ, and QZ geometries.

**Table 2:** Theoretical Equilibrium Geometry and Electric Dipole Moment of YbC_2_

SFX2C-1e-CCSD(T)	*r*(C–C) (Å)	*r*(Yb–C_2_) (Å)	μ (D)
cc-p(w)CVDZ	1.2965	2.1883	10.25
cc-p(w)CVTZ	1.2759	2.1685	10.36
cc-p(w)CVQZ	1.2718	2.1592	10.40
CBS[D-Q]^a^	1.2707	2.1511	

a Complete basis-set estimate by exponential extrapolation of the DZ, TZ, and QZ geometries.

**Table 3:** Theoretical Equilibrium Geometry and Electric Dipole Moment of CaO_2_

CCSD(T)	*r*(O–O) (Å)	*r*(Ca–O_2_) (Å)	μ (D)
cc-pCVDZ	1.5889	1.8467	10.70
cc-pCVTZ	1.5498	1.8212	10.57
cc-pCVQZ	1.5428	1.8157	10.55
cc-pCV5Z	1.5390	1.8157	10.61
CBS[T-5]^a^	1.5345	1.8157	
Δ[*T*(Q)_Λ_ – (T)]^b^	+0.0066	+0.0016	
Best *r*_*e*_ estimate^c^	1.5411	1.8173	

a Complete basis-set estimate by exponential extrapolation of the TZ, QZ, and 5Z geometries.

b The difference between valence-only CCSD­(T) and CCSDT­(Q)_Λ_ geometries using the cc-pCVTZ basis set for Ca and cc-pVTZ basis set for C (with the appropriate orbitals dropped from the correlation treatment).

c The sum of the CBS extrapolation and Δ­[*T*(Q)_Λ_ – (T)] correction.

The equilibrium rotational constants derived from the optimized geometries were further corrected for rovibrational effects based on nuclear motion calculations performed with the NITROGEN package.^37^ Quasi-variational calculations using a numerically exact kinetic energy operator of rovibrational levels with *J* ≤ 3 were performed on potential energy surfaces (PESs) represented as eighth-order power series in internal coordinates. The PESs for CaO_2_ [CCSD­(T)/cc-pCVQZ], SrC_2_ [CCSD­(T)/cc-pCVQZ­(-PP)], and YbC_2_ [SFX2C-1e-CCSD­(T)/cc-p­(w)­CVTZ] were derived from 590, 812, and 736 single-point energies sampled near their equilibrium geometries with rms fit residuals of 0.44, 0.15, and 1.36 cm^–1^, respectively. The theoretical rovibrational correction to a given microwave transition frequency was calculated by the difference between the quasi-variational rovibrational transition frequency and the rigid-rotor transition frequency derived from the equilibrium moments of inertia for the same potential energy surface. The small corrections from the rotational *g*-tensor were neglected. Further details on the rovibrational calculations can be found in our recent studies of MgC_2_ ^11^ and CaC_2_,^12^ in which the same rovibrational methods were applied.

## Results

### Strontium Dicarbide

SrC_2_ is predicted to be a near-prolate T-shaped asymmetric top with *C*_2*v*_ symmetry based on the theoretical structural calculations in this work, which are consistent with prior quantum chemical studies.^3^ Its structure is illustrated in Figure 1 using the best-estimate theoretical geometry derived from the ECP-CCSD­(T) complete basis set extrapolation in Table 1. The two equivalent carbon atoms lead to bosonic nuclear spin degeneracies for Sr^12^C_2_ isotopologues equal to 1 and 0 for rovibrational levels with even and odd values of *K*_*a*_ + *v*_3_, respectively, where *K*_*a*_ is the usual asymmetric top rotational quantum number and *v*_3_ is the number of quanta in the ν_3_ antisymmetric (*b*_2_) bending mode. The fermionic spin degeneracies for Sr^13^C_2_ are instead 1 and 3 for even and odd *K*_*a*_ + *v*_3_, respectively. (The same bosonic spin statistics hold for all M^12^C_2_, M^16^O_2_, and M^18^O_2_ isotopologues that follow. The fermionic statistics for M^17^O_2_ are 15:21 due to the *I* = 5/2 nuclear spin of ^17^O.)

**Figure 1:**
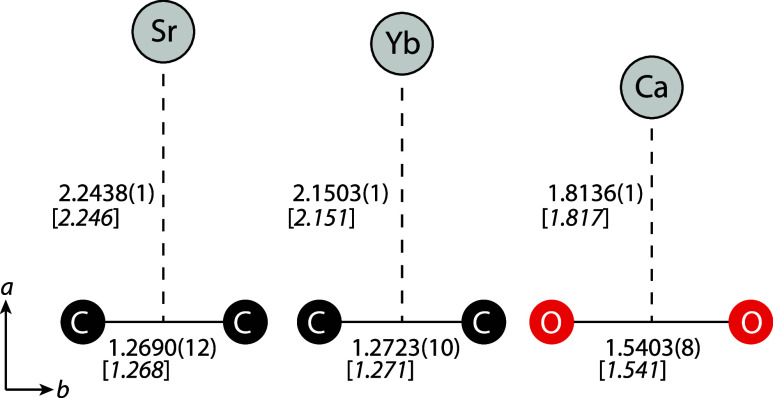
Theoretical (italic, in brackets) and semiexperimental (regular) equilibrium structures of SrC_2_, YbC_2_, and CaO_2_. All bond lengths are given in Å, with 2σ statistical uncertainties shown in parentheses in units of the last digits. The *a* and *b* principal axes are indicated.

After optimizing the experimental conditions for the production of CaC_2_,^12^ we began a search for the two lowest-frequency *a*-type rotational transitions of SrC_2_, *J*_*K*_*a*_ *K*_*c*__ = 1_01_–0_00_ and 2_02_–1_01_, predicted to lie near 10083 and 20162 MHz, respectively. A pair of lines was found at 10106 and 20207 MHz, each only 0.2% higher than the predicted frequencies. Both lines passed a series of experimental assays to test whether their carrier was consistent with SrC_2_. The dependence of the signal on the ablation laser, swapping HCCH for DCCD in the precursor mixture, and external magnetic fields indicated that the carrier contained Sr and no H and had a closed-shell electronic state. DR measurements established that the 10 and 20 GHz transitions shared a common intermediate level and also yielded the detection of the 3_03_–2_02_ transition near 30301 MHz. Definitive confirmation of the assignment was provided by the observation of satellite transitions from six additional isotopic species (Table 4), each of which was detected within 0.2% of the predicted isotopic shift derived from the theoretical equilibrium geometry and vibrational corrections. The ^87^Sr^12^C_2_ isotopologue exhibited a well-resolved hyperfine structure resulting primarily from nuclear electric quadrupole coupling of the ^87^Sr nuclear spin (*I* = 9/2). The hyperfine-resolved transition frequencies are given in Table 5.

**Table 4:** Microwave Transition Frequencies of SrC_2_ in the *v*_3_ = 0 and *v*_3_ = 2 Vibrational States^a^

	*v*_3_ = 0			*v*_3_ = 2		
isotope	1_01_–0_00_	2_02_–1_01_	3_03_–2_02_	1_01_–0_00_	2_02_–1_01_	3_03_–2_02_
^88^Sr^12^C_2_	10105.621 (−51.011)	20207.276 (−102.146)	30300.983* (−153.527)	9985.882	19967.532	29940.715*
^87^Sr^12^C_2_	[10129.367]^b^ (−51.147)	[20254.730]^b^ (−102.419)		[10009.410]^b^	[20014.550]^b^	
^86^Sr^12^C_2_	10153.730 (−51.287)	20303.414 (−102.699)	30445.015*(−154.361)	10033.561	20062.809	30083.426
^88^Sr^12^C^13^C	9783.634 (−48.401)	19563.351 (−96.919)		9669.990	19335.816	28993.314*
^88^Sr^13^C_2_	9487.445 (−46.097)	18970.976 (−92.308)		9379.870	18755.574	28122.953*
^86^Sr^12^C^13^C	9831.709 (−48.671)	19659.425 (−97.461)				
^86^Sr^13^C_2_		19066.985 (−92.839)				

a All values are in MHz. The measurement uncertainty is 2 kHz, except for DR measurements (marked with *), which have an uncertainty of 10 kHz. The calculated rovibrational correction for each *v* = 0 frequency is shown in parentheses.

b Derived hyperfine-free transition frequency. See Table 5 for hyperfine-resolved transition frequencies.

**Table 5:** Hyperfine-Resolved Rotational Transition Frequencies, Nuclear Electric Quadrupole Coupling Constants (*χ*), and Nuclear Spin-Rotation Constants (*C*) of ^87^SrC_2_

*J*_*K*_*a*_ *K*_*c*__^′^ – *J*_*K*_*a*_ *K*_*c*__^″^	*F*′ – *F*″	*v*_3_ = 0^a^	*v*_3_ = 2^a^	theory^b^
1_01_ – 0_00_	4.5 – 4.5	10122.228	10002.496	
	5.5 – 4.5	10132.043	10012.009	
	3.5 – 4.5	10134.282	10014.177	
2_02_ – 1_01_	4.5 – 3.5	20245.353	20005.473	
	5.5 – 5.5	20247.590	20007.636	
	4.5 – 5.5	20247.590	20007.636	
	3.5 – 3.5	20250.141	20010.112	
	6.5 – 5.5	20255.876	20015.669	
	2.5 – 3.5	20256.840	20016.606	
	5.5 – 4.5	20257.406	20017.153	
	4.5 – 4.5	20257.406	20017.153	
	3.5 – 4.5	20262.194	20021.793	
χ_aa_		–53.558(7)	–51.904(8)	–49.0
χ_bb_		24.2(10)	23.2(8)	20.0
$\frac{C_{\mathrm{bb}}+C_{\mathrm{cc}}}{2}\times 10^{3}$		–0.77(17)	–0.66(19)	

a All values are in MHz. The measurement uncertainty is 2 kHz. The 1σ statistical uncertainty of the derived χ and *C* hyperfine parameters is shown in parentheses in units of the last digit.

b Equilibrium nuclear electric quadrupole coupling constants calculated at the SFX2C-1e-CCSD­(T)/cc-p­(w)­CVQZ-X2C level of theory.

The ^87^Sr hyperfine splittings could be reproduced with the SPFIT program^38^ to within the measurement uncertainty with an effective hyperfine Hamiltonian that included the nuclear electric quadrupole coupling (χ_aa_, χ_bb_, and χ_*cc*_, assuming χ_aa_ + χ_bb_ + χ_*cc*_ = 0) as well as a single linear combination of nuclear spin-rotation coupling terms [(*C*_bb_ + *C*_cc_)/2, which is the only well-determined linear combination for the rotational transitions measured].

The limited number of rotational transitions measured for each isotopic species did not permit determination of all three rotational constants independently. Nonetheless, the molecular structure could still be derived directly from the transition frequencies, given the large number of isotopic variants. We derived the semiexperimental equilibrium geometry of SrC_2_ by fitting the structural parameters to the 15 ground-state measured frequencies in Table 4 corrected by the theoretical difference between the ground-state and equilibrium values (shown in parentheses for each transition in Table 4). The best-fit geometry and frequency residuals are shown in Table 6.

**Table 6:** Semiexperimental Equilibrium Geometries of SrC_2_, YbC_2_, and CaO_2_

parameter^a^	SrC_2_	YbC_2_	CaO_2_
*r*(X–X)/Å	1.2690(12)	1.2723(10)	1.5403(8)
*r*(M–X_2_)/Å	2.2438(1)	2.1503(1)	1.8136(1)
RMSE/MHz^b^	0.031	0.014	0.144

a The 2σ statistical uncertainties are shown in parentheses in units of the last digit. X = C or O. *r*(M–X_2_) is the distance from the metal to the X_2_ midpoint.

b The root-mean-square fit error of the semiexperimental equilibrium transition frequencies derived from the *v* = 0 measurements and theoretical rovibrational corrections reported in Tables 4, 8, and 11.

In the course of the cavity FTMW searches, additional microwave transitions were observed that had the expected Sr-isotope frequency shift patterns and passed the same laboratory assays as those for SrC_2_ listed above. It was ultimately established that these molecular features were in fact vibrational satellites of SrC_2_, which we assigned to a series of overtones of the ν_3_ antisymmetric bending mode (*b*_2_ symmetry) by using the vibrational shifts predicted by our quasi-variational rovibrational calculations. The most extensive set of microwave transitions, including isotopic species, were measured for the *v*_3_ = 2 overtone level and are listed in Table 4.

Additional vibrational satellites of ν_3_ overtones were detected up to *v*_3_ = 6, which has a theoretical vibrational energy of 2300 cm^–1^. As discussed above, vibrational states with even values of *v*_3_ have rotational levels with even *K*_*a*_ only, while states with odd values of *v*_3_ have odd *K*_*a*_, owing to the bosonic nuclear spin statistics of the ^12^C nuclei. The microwave transition frequencies of the lowest two transitions with even or odd *K*_*a*_ values were within the spectrometer bandwidth and are listed in Table 7. The cavity spectra of the 1_01_–0_00_ transitions for the *v*_3_ = 0, 2, 4, and 6 vibrational levels are shown in Figure 2a. Assuming the rotational temperature in each state to be the same, we derive an effective vibrational temperature of the ν_3_ mode to be 1210 ± 120 K (Figure 2b). The ν_1_ (CC stretch, 1756 cm^–1^) and ν_2_ (Sr–C_2_ stretch, 462 cm^–1^) vibrational modes appear to be out of equilibrium with the ν_3_ bending mode, i.e., significantly colder. At the effective vibrational temperature of ν_3_, the ν_1_ and ν_2_ vibrational satellites should be detectable, but modest searches for them based on the predicted vibrational shifts were unsuccessful, with upper limits of their populations equal to ∼2% of that of the vibrational ground state.

**Figure 2:**
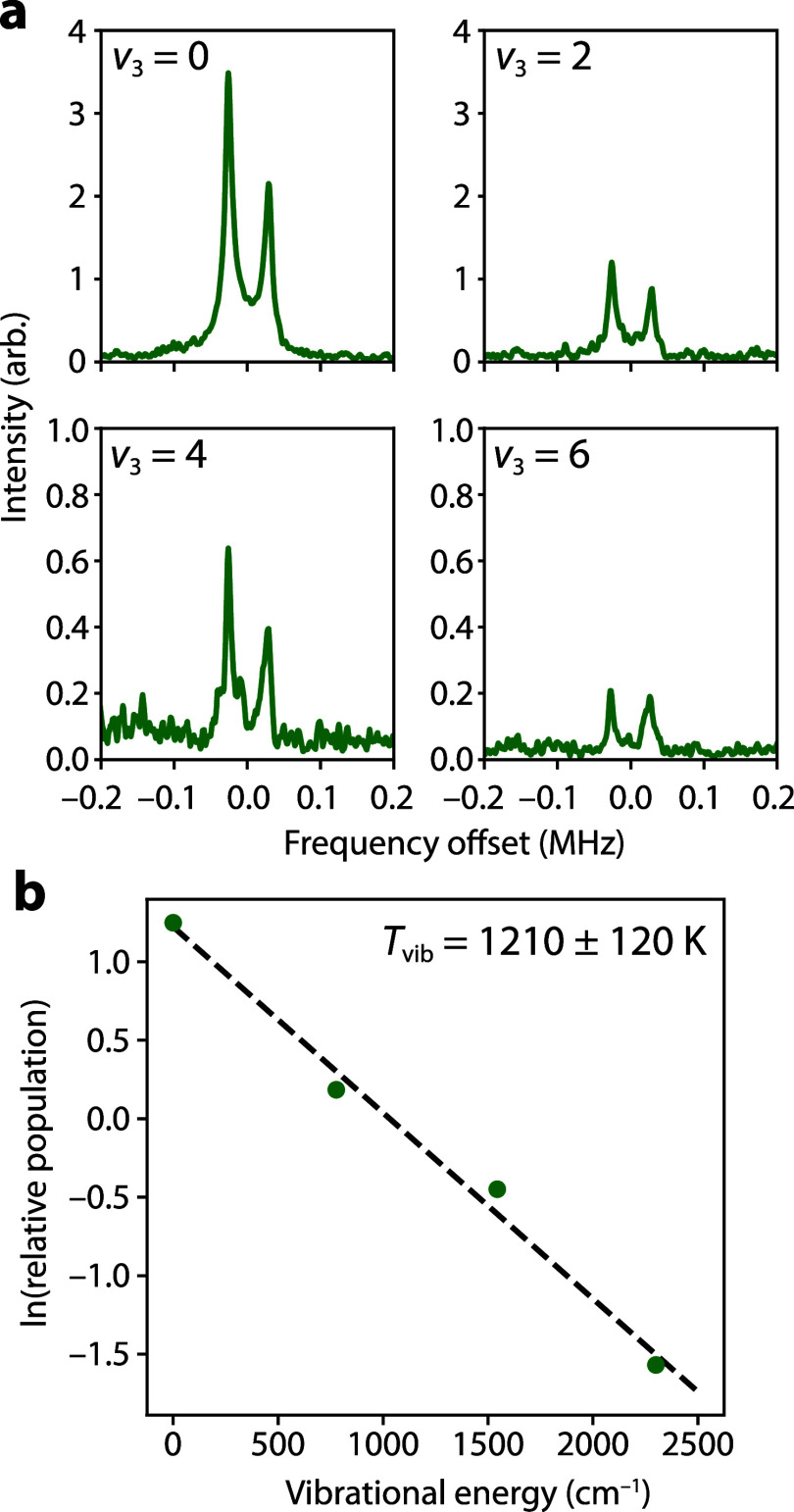
Vibrationally excited states of ^88^Sr^12^C_2_. The 1_01_–0_00_ rotational transitions are shown in (a) for the *v*_3_ = 0, 2, 4, and 6 vibrational levels with a common vertical scale. The horizontal axis of each panel is the frequency offset relative to the rest transition frequencies in Table 7. In (b), the relative vibrational intensities are plotted against the calculated vibrational energies to derive an effective vibrational temperature of *T*_vib_ = 1200 ± 120 K.

**Table 7:** Microwave Transition Frequencies of the *v*_3_ = 0–6 Vibrational States of ^88^SrC_2_

*v*_3_	*E*_vib_ (cm^–1^)^a^	*J*_*K*_*a*_ *K*_*c*__^′^ – *J*_*K*_*a*_ *K*_*c*__^″^ ^b^		
		1_01_–0_00_	2_02_–1_01_	Obs.–Calc.^c^
0	0	10105.621	20207.276	
2	777	9985.882	19967.532	–0.12
4	1544	9869.898	19735.316	–0.20
6	2300	9757.829	19510.942	–0.12
		2_12_–1_11_	2_11_–1_10_	Obs.–Calc.^d^
1	390	19582.207	20597.937	
3	1162	19323.657	20384.223	1.09
5	1923	19072.797	20176.858	5.41

a The calculated vibrational energy.

b All frequencies are in MHz. The measurement uncertainty is 2 kHz.

c The difference between the observed and calculated vibrational shift of the 1_01_ – 0_00_ transition relative to *v*_3_ = 0.

d The difference between the observed and calculated vibrational shift of the 2_12_ – 1_11_ transition relative to *v*_3_ = 1.

### Ytterbium Dicarbide

The structure and spectroscopy of YbC_2_ are very similar to those of SrC_2_, and its laboratory microwave detection proceeded similarly. The 1_01_–0_00_ and 2_02_–1_01_ transitions of the most abundant isotopologue, ^174^Yb^12^C_2_, were predicted to lie at 9839.9 and 19676.3 MHz based on the best-estimate theoretical equilibrium geometry (Table 2) and zero-point corrections. After a brief cavity FTMW search, two lines were observed at 9846.1 and 19688.6 MHz, only 0.06% above the prediction. They satisfied the same experimental assays as described above for SrC_2_ and were tentatively assigned to ^174^Yb^12^C_2_. Satellite lines for ^170^Yb, ^171^Yb, ^172^Yb, ^173^Yb, and ^176^Yb were subsequently detected in natural abundance at the expected frequency shift and intensity relative to the major isotopic species on the assumption that the carrier was YbC_2_. Additional measurements with a ^13^C-enriched C_2_H_2_ precursor mixture yielded 3 single-^13^C-substituted isotopologues and 2 double-^13^C-substituted isotopologues (Table 8).

**Table 8:** Microwave Transition Frequencies of YbC_2_

isotope	1_01_–0_00_ ^a^		2_02_–1_01_ ^a^	
^176^Yb^12^C_2_	9833.095	(−49.935)	19662.608	(−99.978)
^174^Yb^12^C_2_	9846.098	(−50.010)	19688.596	(−100.130)
^172^Yb^12^C_2_	9859.397	(−50.088)	19715.173	(−100.286)
^170^Yb^12^C_2_	9873.001	(−50.167)	19742.361	(−100.445)
^176^Yb^13^C^12^C	9484.099	(−47.179)	18964.715	(−94.461)
^174^Yb^13^C^12^C	9497.097	(−47.253)	18990.695	(−94.610)
^172^Yb^13^C^12^C	9510.392	(−47.329)	19017.263	(−94.762)
^174^Yb^13^C_2_	9176.231	(−44.820)	18349.014	(−89.738)
^172^Yb^13^C_2_	9189.520	(−44.895)	18375.569	(−89.887)

a All values are in MHz, and the measured frequencies have an uncertainty of 2 kHz. The quantity in parentheses is the theoretical ground-state rovibrational correction for each transition frequency calculated as described in the text.

Nuclear electric quadrupole and spin-rotation hyperfine splittings were resolvable in the odd isotopes of Yb owing to the nonzero nuclear spin of ^173^Yb (*I* = 5/2) and ^171^Yb (*I* = 1/2). Their microwave transitions and derived hyperfine coupling constants are given in Table 9. The ^173^Yb nuclear electric quadrupole coupling constants (χ_aa_ and χ_bb_) are in good agreement with the predictions derived from the SFX2C-1e-CCSD­(T) electric field gradients. Although we do not have theoretical predictions of the nuclear spin-rotation constants (*C*_*ii*_, which are a second-order property), the ratio of the observed values of (*C*_bb_ + *C*_c*c*_) /2 for ^173^Yb and ^171^Yb, −3.3(2)/11.7(16) ≈ −0.28(4), agrees well with the ratio of the nuclear *g* factors, −0.26,^39^ as expected. Finally, the semiexperimental equilibrium geometry of YbC_2_ was derived using the same procedure as SrC_2_, and the best-fit geometrical parameters are listed in Table 6.

**Table 9:** Hyperfine-Resolved Rotational Transition Frequencies, Nuclear Electric Quadrupole Coupling Constants (*χ*), and Nuclear Spin-Rotation Constants (*C*) of ^173^YbC_2_ and ^171^YbC_2_

*J*_*K*_*a*_ *K*_*c*__^′^ – *J*_*K*_*a*_ *K*_*c*__^″^	*F*′ – *F*″	^173^YbC_2_ ^a^	theory^b^
1_01_ – 0_00_	2.5 – 2.5	9629.743	
	3.5 – 2.5	9924.024	
	1.5 – 2.5	10051.255	
2_02_ – 1_01_	2.5 – 1.5	19407.277	
	3.5 – 3.5	19461.584	
	2.5 – 3.5	19534.511	
	1.5 – 1.5	19605.830	
	4.5 – 3.5	19732.384	
	3.5 – 2.5	19755.866	
	0.5 – 1.5	19786.460	
	2.5 – 2.5	19828.792	
	1.5 – 2.5	20027.345	
χ_aa_		–1402.993(5)	–1350.3
χ_bb_		573.1(6)	544.4
$\frac{C_{\mathrm{bb}}+C_{\mathrm{cc}}}{2}\times 10^{3}$		–3.3(2)	
*J*′_*K*_*a*_ *K*_*c*__ – *J*″_*K*_*a*_ *K*_*c*__	*F*′ – *F*″	^171^YbC_2_ ^a^	
1_01_–0_00_	0.5 – 0.5	9866.142	
	1.5 – 0.5	9866.160	
2_02_–1_01_	1.5 – 0.5	19728.672	
	2.5 – 1.5	19728.683	
$\frac{C_{\mathrm{bb}}+C_{\mathrm{cc}}}{2}\times 10^{3}$		11.7(16)	

a All values are in MHz. The measurement uncertainty is 2 kHz. The 1σ statistical uncertainty of the derived χ and *C* hyperfine parameters is shown in parentheses in units of the last digit.

b Equilibrium nuclear electric quadrupole coupling constants calculated at the SFX2C-1e-CCSD­(T)/cc-p­(w)­CVQZ-X2C level of theory.

Only a cursory search for excited vibrational states of YbC_2_ was performed. Satellites were observed near the expected positions for the *v*_3_ = 2 and 4 overtones. However, the measured frequencies (Table 10) appeared approximately halfway between the predicted positions for the vibrational satellites of ^174^YbC_2_ and ^172^YbC_2_. Owing to the relatively narrow search window (∼14 MHz), we cannot therefore definitively assign the Yb isotope carrier. More comprehensive cavity FTMW measurements of vibrationally excited YbC_2_ require future experiments.

**Table 10:** Microwave Transition Frequencies of the *v*_3_ = 0, 2, and 4 Vibrational States of YbC_2_

*v*_3_	*E*_vib_ (cm^–1^)^a^	*J*_*K*_*a*_ *K*_*c*__^′^– *J*_*K*_*a*_ *K*_*c*__^″^ ^b^		
		1_01_–0_00_	2_02_–1_01_	Obs.–Calc.^c^
0	0	9846.098	19688.596	
2	828	9716.977	19430.051	7.96
4	1643	9594.289	19084.403	13.8

a The calculated vibrational energy.

b All frequencies are in MHz. The measurement uncertainty is 2 kHz.

c The difference between the observed and calculated vibrational shift of the 1_01_ – 0_00_ transition relative to *v*_3_ = 0, *v*_3_ = 2 and 4 lines assuming they are carried by ^174^Yb^12^C_2_.

### Calcium Peroxide

Owing to the lighter mass and shorter metal–ligand bond length of CaO_2_ (Figure 1), only one of its rotational transitions was expected in the frequency range of the cavity spectrometer. After a search centered near the best-estimate zero-point-corrected prediction for the fundamental 1_01_–0_00_ transition frequency (15065.9 MHz), an unassigned line was detected at 15123.586 MHz, about 0.4% higher than the prediction. As with SrC_2_ and YbC_2_, experimental tests confirmed the carrier of this line contained calcium, oxygen, and had a closed-shell electronic state, and it was tentatively assigned to ^40^Ca^16^O_2_. Additional searches near the isotopically shifted frequencies predicted based on the tentative ^40^Ca^16^O_2_ assignment yielded detections of the 1_01_–0_00_ transitions of ^40^Ca^16^O^18^O, ^40^Ca^18^O_2_, and ^40^Ca^17^O_2_, at frequencies within 0.2% of the predicted isotopic shift. We also measured the 2_02_–1_01_ transition of the parent isotopologue via DR at 30066.939 MHz, within 0.017% of the position predicted by scaling the purely theoretical value by the ratio of the theoretical and measured 1_01_–0_00_ transition frequencies. These measurements are summarized in Table 11. (No attempt was made to detect vibrational satellites of CaO_2_.)

**Table 11:** Microwave Transition Frequencies of CaO_2_

isotope	1_01_–0_00_ ^a^		2_02_–1_01_ ^a^	
^40^Ca^16^O_2_	15123.586	(−53.516)	30066.939*	(−110.707)
^40^Ca^16^O^18^O	14581.582	(−50.463)		
^40^Ca^18^O_2_	14108.311	(−47.807)		
^40^Ca^17^O_2_	14585.496	(−50.467)		

a All values are in MHz. The measured frequencies have an uncertainty of 2 kHz, except for the DR measurement (marked with *), which has an uncertainty of 10 kHz. The quantity in parentheses is the theoretical ground-state rovibrational correction for each transition frequency calculated as described in the text.

Although no additional isotopic species were detected because of the low natural abundance of heavier Ca isotopes (≤2%), the present data are still sufficient for a semiexperimental equilibrium structure determination using the same approach as above. The best-fit values of the two geometrical parameters derived from the 5 transitions and vibrational corrections listed in Table 11 are presented in Table 6.

### Vibrationally Excited Calcium Dicarbide

Motivated by the facile vibrational excitation observed for SrC_2_ and YbC_2_ in the ablation–discharge expansion source, we performed additional experiments to detect vibrational satellites of CaC_2_, the ground-state microwave spectrum of which was recently detected in our laboratory and in space.^12^ We measured the 1_01_–0_00_ transition of several excited states up to ∼1800 cm^–1^ of vibrational energy, each of which was within 1–2 MHz of the prediction based on our prior rovibrational calculations (Table 12). The ν_1_ (CC stretch), ν_2_ (Ca–C_2_ stretch), and 2ν_2_ excited states were observed in addition to the anticipated ν_3_ bending overtones. The cavity spectrometer frequency range and ^12^C spin statistics limited these measurements to only the even-*v*_3_ vibrational levels. The vibrational Boltzmann plot in Figure 3 demonstrates that the vibrational excitation in CaC_2_ is qualitatively similar to that in SrC_2_. The ν_3_ mode has a high effective vibrational temperature, *T*_vib_ = 840 ± 130 K, while the ν_1_ and ν_2_ stretching modes are significantly colder.

**Figure 3:**
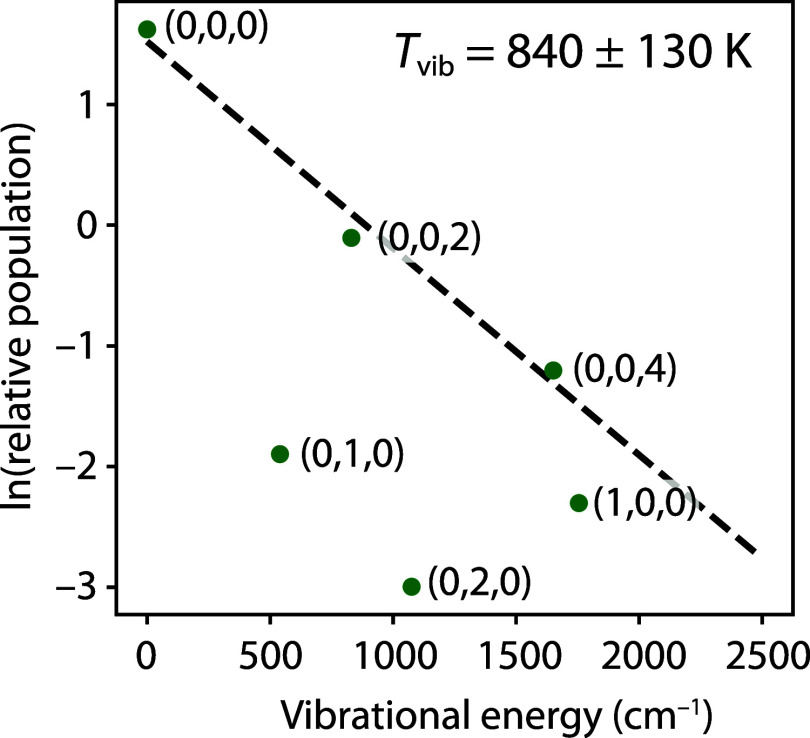
Vibrational excitation of CaC_2_. The effective ν_3_ temperature (840 ± 130 K) was derived from fitting only the *v*_3_ = 0, 2, and 4 states (dashed line). Each point is labeled by (*v*_1_,*v*_2_,*v*_3_) vibrational quantum numbers.

**Table 12:** *J*_*K*_*a*_ *K*_*c*__ = 1_01_–0_00_ Transition Frequency for Vibrationally Excited States of ^40^CaC_2_

*v*_1_	*v*_2_	*v*_3_	*E*_vib_ ^a^ (cm^–1^)	frequency^b^ (MHz)	Obs.–Calc.^c^ (MHz)
0	0	0	0	14134.168	
0	1	0	540	14070.222	0.81
0	0	2	830	13963.951	0.96
0	2	0	1075	14005.125	1.69
0	0	4	1651	13799.617	0.68
1	0	0	1755	14129.661	0.03

a The calculated vibrational energy.

b The measured transition frequencies have an uncertainty of 2 kHz.

c The difference between the observed and theoretically predicted vibrational shift.

## Discussion

*Metal dicarbides*—Now that the semiexperimental equilibrium geometries are known from precise microwave data of several alkaline earth­(-like) dicarbides, it is possible to compare the chemical trends of their structures. The heavy species reported in this work, SrC_2_ and YbC_2_, have the same T-shaped configuration as the lighter alkaline earth dicarbides BeC_2_,^8^ MgC_2_,^11^ and CaC_2_.^12^ The C–C bond lengths are remarkably insensitive to the metal cation, differing by less than 0.004 Å. The metal–ligand bond lengths have a much wider distribution, which closely tracks the changes in the metal ionic radius inferred from the structures of ionic solids. For example, Figure 4 shows that the differences in the molecular M–C_2_ bond lengths have a nearly 1:1 relationship with the corresponding differences in the nominal M^2+^ ionic radii. The vertical intercept of the M–C_2_ bond length versus the M^2+^ ionic radius is approximately equal to 1 Å, which may be interpreted as the effective ionic radius of C_2_^2–^ perpendicular to the C–C bond. The M–O oxides show a qualitatively similar trend, but with a smaller slope between the molecular and solid-state structures. The semiexperimental structures and large calculated dipole moments of SrC_2_ and YbC_2_ (>10 D) lend strong evidence for describing them as having nominal M^2+^L^2–^ ionic bonding.

**Figure 4:**
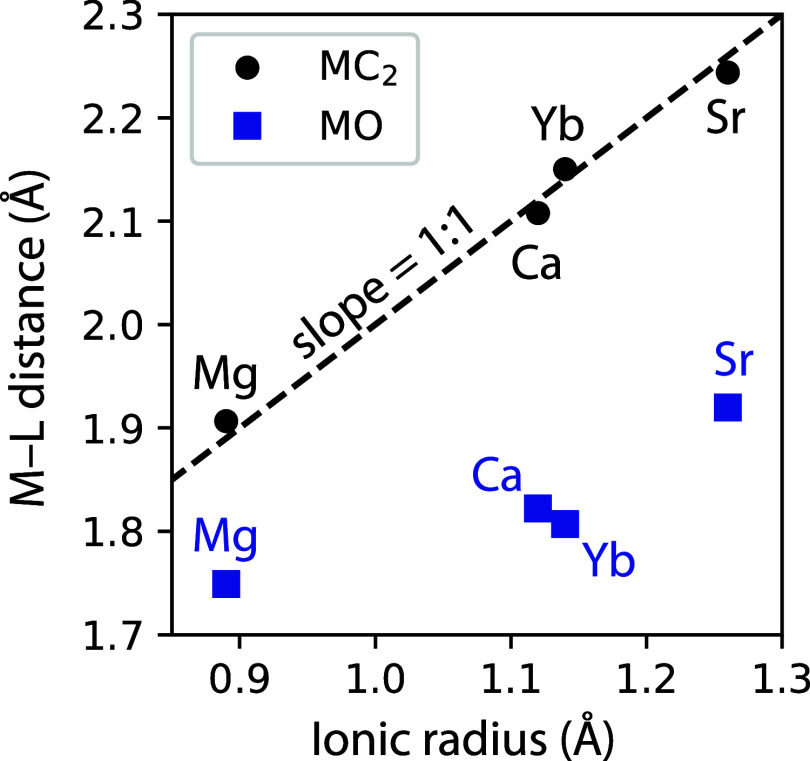
Metal–ligand bond lengths in oxide and pseudo-oxide molecules. The vertical axis is the metal–ligand bond length derived for the molecular monomers. The horizontal axis is the nominal ionic radius for +2 charge (VIII coordination) tabulated in ref 40. The molecular metal oxide equilibrium bond lengths were derived from refs 41–44 .

The highly nonequilibrated vibrational temperatures observed for CaC_2_, SrC_2_, and YbC_2_ are typical of small diatomic and triatomic species in supersonic discharge expansions. Prior cavity FTMW experiments of SO, SiO, and SiS produced with a similar discharge nozzle and source conditions as that used here reported effective vibrational temperatures up to 10^4^ K,^45^ which is approximately equal to the electron temperature of the discharge plasma. A detailed examination of the vibrational excitation mechanisms of the metal dicarbides studied here is beyond the scope of this paper but would likely be a fruitful direction for future work.

*Calcium peroxide*—Cryogenic matrix infrared measurements of various calcium oxides produced by pulsed laser ablation have identified at least two stable isomers of CaO_2_: a singlet ^1^A_1_ T-shaped (or cyclic) CaO_2_ peroxide species and a triplet ^3^B_2_ open bent OCaO dioxide species, which is approximately 10 kcal/mol higher in energy.^4,46^ The CaO_2_ species whose microwave spectrum we have detected is clearly the ^1^A_1_ peroxide given its structure and observed spin multiplicity.

The rotational spectra of MO_2_ molecules are known for only a small number of other examples, including TiO_2_,^16^ ZrO_2_,^47^ and HfO_2_.^48^ These Group 4 transition metals are all metal dioxides with nominal O^–^M^2+^O^–^ bonding. This classification is borne out by the long O–O distances (ca. 2.8 Å) in these dioxides relative to the much shorter value in CaO_2_ (1.5403(8)­Å, Table 6). It appears that CaO_2_ is the only metal peroxide for which precise rotational data are available. Isolated O_2_^2–^ peroxide molecules cannot be studied with high-resolution spectroscopy in the gas phase because they are unstable to spontaneous electron detachment. The structure of the intramolecular O_2_^2–^ ligand therefore provides a unique proxy for comparison to the structures of gas-phase O_2_, its singly charged ions, and other peroxides.

Table 13 summarizes the experimental bond length information available for such reference species containing an O–O bond. If we take the O_2_^2–^ ligand in CaO_2_ as a proxy for the free dianion, then there is a clear trend in bond order vs bond length in the O_2_^+^, O_2_, O_2_^–^, and O_2_^2–^ species. This “dianion-in-molecule” approach is reminiscent of (if somewhat cruder than) that used by Baldwin et al. to derive the negative electron affinity of O^–^ (i.e., O^–^ + e^–^ → O^2–^) via a diabatic analysis of CaO potential energy curves.^49^

**Table 13:** Measured Bond Lengths of Charged and Neutral Species Containing an O–O Bond

species	formal bond order	r(O–O)^a^ (Å)
O_2_^+^	2.5	1.1164(4)^b^
O_2_	2.0	1.20748(4)^c^
O_2_^–^	1.5	1.346(8)^d^
Ca^2+^O_2_^2–^	1.0	1.5403(8)^e^
H_2_O_2_	1.0	1.4524(6)^f^
Cl_2_O_2_	1.0	1.426(4)^g^
F_2_O_2_	1.0	1.217(6)^h^

a The estimated 2σ uncertainty is given in parentheses in units of the last digit.

b *r*_eq_ from ref 50.

c *r*_eq_ from ref 51.

d Weighted average of *r*_*eq*_ derived from the Franck–Condon analysis of refs 52–54 .

e Semiexperimental *r*_eq_, this work.

f Semiexperimental *r*_eq_ from ref 55.

g *r*_0_ from ref 56.

h Ground-state substitution structure from ref 57.

It is also instructive to compare the O_2_^2–^ ligand to nominal single O–O bonds in other peroxides. The single-bond length in H_2_O_2_ is significantly shorter (∼0.1 Å) than CaO_2_, which suggests that the excess negative charge in CaO_2_ lengthens the bond as would be expected from the combined contributions of greater antibonding π* orbital occupancy and simple Coulomb repulsion. Indeed, when H is replaced by the more electron-withdrawing Cl in chlorine peroxide, which removes electron density from the O_2_ fragment, the single-bond length of the O–O bond decreases. F_2_O_2_ extends this trend even further, with the shortest O–O “single”-bond length in Table 13. Figure 5 provides a more quantitative demonstration of this overall trend by correlating the O_2_ bond length with the total Mulliken charge of the O_2_ unit.

**Figure 5:**
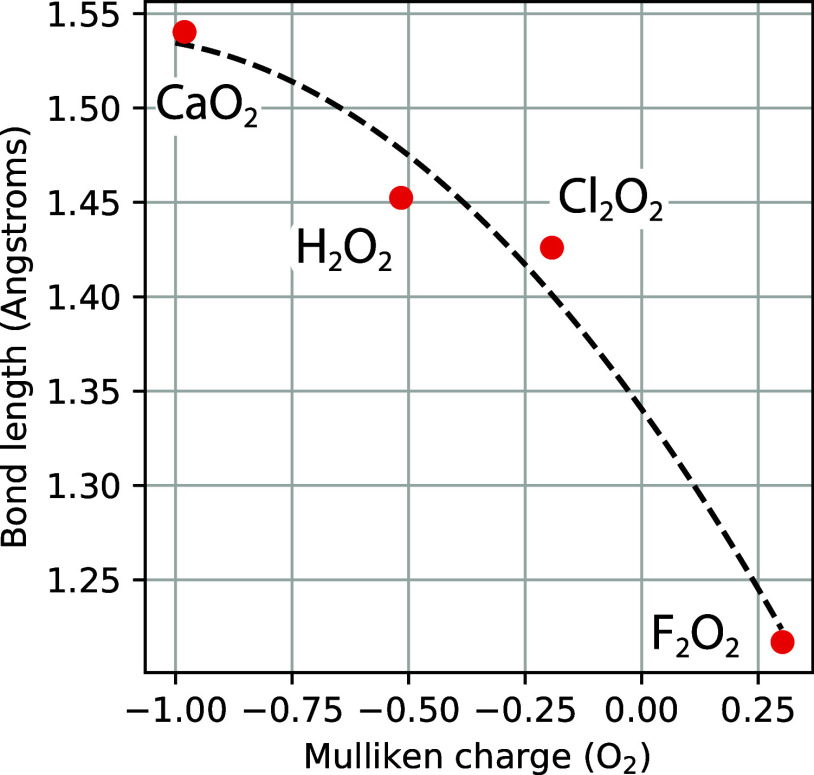
O–O bond length of several peroxides compared to partial charge of the O_2_ fragment. The red circles are from bond length data in Table 13 and Mulliken charges calculated with CCSD­(T)/cc-pCVQZ for CaO_2_ and CCSD­(T)/cc-pVQ­(+d)­Z for H_2_O_2_, F_2_O_2_, and C_2_O_2_ (i.e., including “+d” tight d functions added for Cl^58,59^ ). The black dashed trace is a quadratic trend line.

*Astrochemical prospects*—Our recent studies of the microwave rotational spectra of MgC_2_ and CaC_2_ led to their discovery in the circumstellar envelope (CSE) of the evolved carbon-rich star IRC+10216.^11,12^ Because of their similar valence electronic structure, gas-phase Sr and Yb present in this CSE likely undergo chemistry similar to that of Mg and Ca, i.e., storing a significant fraction of their gas-phase abundance in MC_2_ dicarbides. However, given the much smaller total abundance of Sr and Yb relative to Mg and Ca (several orders of magnitude^60^), the detection of SrC_2_ and YbC_2_ in IRC+10216 is likely beyond the current reach of even the most sensitive radio telescopes.

In contrast, CaO_2_ is a plausible candidate for detection in oxygen-rich CSEs, such as that of VY Canis Majoris, which is known to harbor small metal oxides, including TiO, TiO_2_, and AlO.^61,62^ Our precise experimental and theoretical data lay the foundation for its potential radio detection. As only two calcium-bearing molecules, CaNC^63^ and CaC_2_,^12^ are currently known in CSEs, the detection and astrophysical characterization of CaO_2_ would substantially add to our understanding of circumstellar calcium chemistry.

## Conclusions

Our results establish that CaO_2_, SrC_2_, and YbC_2_ are all well described as having dominant M^2+^L^2–^ charge configurations in their ground electronic state. The two dicarbides have equilibrium structures and ionic bonding patterns remarkably similar to those of the lighter alkaline earth metal dicarbides, with metal–ligand bond lengths well correlated to the nominal metal ionic radii. The microwave spectrum of CaO_2_ provides unique insights into O–O bonding in highly reduced O_2_ peroxide units. Future microwave spectroscopy and diabatic analysis of the excited vibrational states of CaO_2_, particularly those leading toward metal–ligand dissociation, may lead to meaningful experimental constraints on the structure of the electronically unstable gas-phase O_2_^2–^ dianion.
